# Descriptions of eleven Opatrini pupae (Coleoptera, Tenebrionidae) from China

**DOI:** 10.3897/zookeys.291.4780

**Published:** 2013-04-17

**Authors:** Jia Long, Ren Guo-Dong, Yu You-Zhi

**Affiliations:** 1 College of Life Sciences, Hebei University, Baoding, 071002, P. R. China; 2 School of Agriculture, Ningxia University, Yinchuan, 750021, P. R. China

**Keywords:** Tenebrionidae, Opatrini, pupa, taxonomy, China

## Abstract

The pupal stage of eleven Opatrini species occuring in the northern China are described and a key for their identifiaction is provided. The species are *Scleropatrum horridum horridum* Reitter, *Gonocephalum reticulatum* Motschulsky, *Opatrum (Opatrum) subaratum* Faldermann, *Eumylada potanini* (Reitter), *Eumylada punctifera* (Reitter), *Penthicus (Myladion) alashanicus* (Reichardt), *Penthicus (Myladion) nojonicus* (Kaszab), *Myladina unguiculina* Reitter, *Melanesthes (Opatronesthes) rugipennis* Reitter, *Melanesthes (Melanesthes) maxima maxima* Ménétriès and *Melanesthes (Melanesthes) jintaiensis* Ren.

## Introduction

Studies of immatures stages of the insect are needed and important due to the fact, that the results are useful for phylogenetic analysis of particular groups which has already been shown many times (e.g. Böving and Craighead 1931; Beutel and Friedrich 2005). However, taxonomic studies on immature stages of the family Tenebrionidae are rather sporadic and therefore our knowledge of such developmental stages is very limited. This especially holds true for tenebrionid pupae, descriptions of which are rather extremely rare. So this is the primary aim for the present study.

For the tenebrionid pupae, several workers have made their contributions (St-George 1924; Daggy 1946; Ho 1960, 1969; Abdulla 1964; Spilman 1966, 1969, 1979, 1984; Watt 1974; Wei et al. 1989; Ren and Ye 1990; Steiner 1995; Bouchard and Steiner 2004; Yu and Yang 2004; Cherney 2006; Gosik 2007; Dunford and Steiner 2007; Simões et al. 2009; Matthews et al. 2010; Purchart and Nabozhenko 2012), but a few involved the tribe Opatrini, including Ogloblin and Kolobova (1927), Wu and Gao (1978), Yu et al. (1993), Yu et al. (1999), Cherney (2005) and Cherney and Fedorenko (2006).

In this study, the pupal stage of eleven Opatrini species occuring in the northern China are described in detail based on the material at our disposal. The species are *Scleropatrum horridum horridum* Reitter, *Gonocephalum reticulatum* Motschulsky, *Opatrum (Opatrum) subaratum* Faldermann, *Eumylada potanini* (Reitter), *Eumylada punctifera* (Reitter), *Penthicus (Myladion) alashanicus* (Reichardt), *Penthicus (Myladion) nojonicus* (Kaszab), *Myladina unguiculina* Reitter, *Melanesthes (Opatronesthes) rugipennis* Reitter, *Melanesthes (Melanesthes) maxima maxima* Ménétriès and *Melanesthes (Melanesthes) jintaiensis* Ren. Each species is provided with photos of habitus, head, pronotum, lateral processes of abdominal tergites V and VII and urogomphi. Also, a key for their identification is provided. Besides, the diagnosis of tribe is summarized.

## Materials and methods

The study was based on the examination of 26 pupal specimens of Opatrini, which were identified as 11 species belonging to 7 genera. All of them were obtained by breeding from adults in the lab. All pupae are preserved in the glycerine and alcohol solution (1: 20) and deposited in the School of Agriculture of Ningxia University, Yinchuan, China.

Terminology of morphological features followed that of Bouchard and Steiner (2004) and Gosik (2007). Abbreviations are used as follows: BL: body length (from the posterior border of abdominal tergite IX to head); PL: pronotal length (from anterior to posterior border); PW: pronotal width (from one lateral border to the other at the maximal point); MSL: mesonotal length (from anterior to posterior border); MTL: metanotal length (from anterior to posterior border); UL: urogomphus length (from the base to apex of urogomphus); BUL: distance between urogomphi (from one apex to the other of urogomphus).

**Figure 1.**
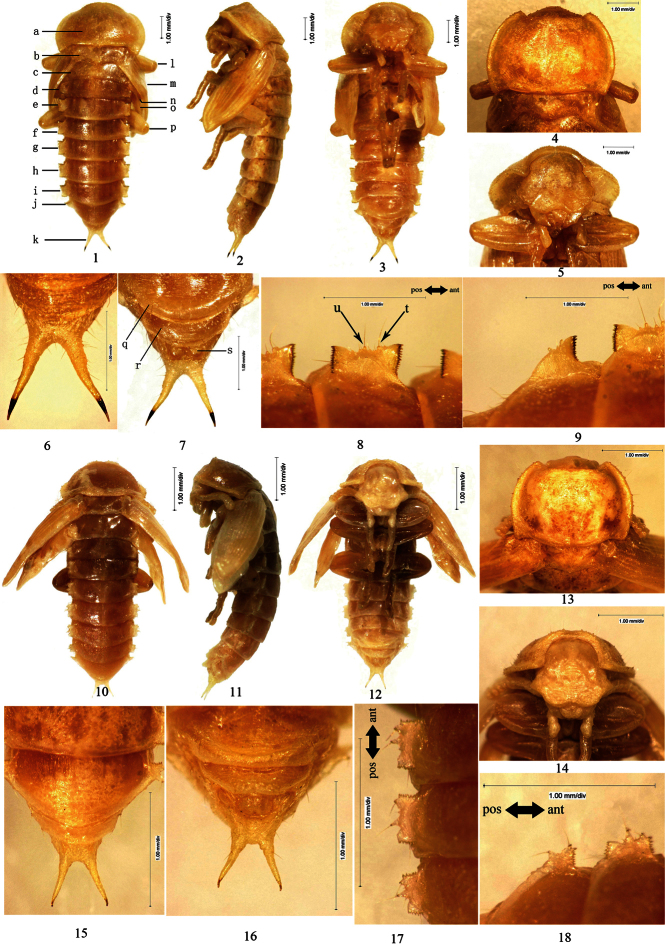
**1–9** *Scleropatrum horridum horridum* Reitter, 1898 **1** Pupal habitus in dorsal view **2** Pupal habitus in lateral view **3** Pupal habitus in ventral view **4** Pronotum **5** Head **6** Urogomphi in dorsal view **7** Urogomphi in ventral view **8** Lateral process of abdominal tergite V **9** Lateral process of abdominal tergite VII **10–18** *Gonocephalum reticulatum* Motschulsky, 1854 **10** Pupal habitus in dorsal view **11 **Pupal habitus in lateral view **12** Pupal habitus in ventral view **13** Pronotum **14** Head **15** Urogomphi in dorsal view **16 **Urogomphi in ventral view **17** Lateral process of abdominal tergite V **18** Lateral process of abdominal tergite VII. **a** Pronotum **b** Mesonotum **c** Metanotum **d** Abdominal segment I **e** Abdominal segment II **f** Abdominal segment III **g** Abdominal segment IV **h** Abdominal segment V **i** Abdominal segment VI **j** Abdominal segment VII **k** Urogomphi **l** Profoot **m** Elytral sheath **n** Mesofoot **o** Metathoracic wing sheath **p** Metafoot **q** Abdominal sternite VII **r** Abdominal sternite VIII **s** Gonotheca **t** large setose tubercle I **u** large setose tubercle II.

The measurements and photographing were carried out under the Free Angle Observation System VHX-100 (Keyence international trade company limited, Japan). When more than one pupa for a taxon was used, the range of values is given. The figures of lateral processes of abdominal tergites in this study are photographed in dorsal view and with the head towards the right. Lateral processes of abdominal tergites I–VII each has 2 large setose tubercles along outer border, of which the anterior one is named as large setose tubercle I (Fig. 1: t), the posterior one as large setose tubercle II (Fig. 1: u).

### Description

*Pupal characteristics of Opatrini* The body white to light yellow (light brown before emergence of imago). Head bent to pronotum (Fig. 1: 2). Anterior border of clypeus emarginated in middle (Fig. 1: 5). Dorsomeson distinct. Pronotal shape as that of adult. Mesonotum slightly convex in middle of posterior part (Fig. 1: 4). Elytral sheath shroud metathoracic wing sheath (Fig. 1: 1–3) completely or not (Fig. 1: 10–12, Fig. 3: 10–12). Apices of elytral sheath acute (Fig. 1: 2–3). Abdominal tergites I–VII each with a pair of lateral processes in middle of lateral borders. Lateral processes dorsoventrally flattened and lune plateform in lateral view, with anterior and posterior borders strongly sclerotized and densely dentated (Fig. 1: 8–9), except weakly sclerotized at anterior borders of those of abdominal tergite I and posterior borders of VII (Fig. 1: 1, Fig. 1: 9). Abdominal tergites I–VI each with a pair of spiracles in front of lateral processes, the spiracles of abdominal tergites II–VI visible in lateral view. Posterior borders of abdominal tergites I–VI straight or broadly emarginated, VII–VIII broadly protuberant. Abdominal tergites I–VIII slightly narrowed posteriorly (Fig. 1: 1), IX with a pair of well-developed urogomphi (Fig. 1: 6–7).

### Key to the known pupae of the tribe Optarini from China

**Table:** 

1	Posterior border of the pronotum bisinuate (Fig. 1: 4, 13; Fig. 2: 4, 13; Fig. 3: 4)	2
–	Posterior border of the pronotum broadly protuberant	6
2	Urogomphi diverging from each other (Fig. 1: 6–7, 15–16; Fig. 2: 6–7)	3
–	Urogomphi parallel to each other (Fig. 2: 15–16; Fig. 3: 6–7) (*Eumylada* Reitter, 1889)	5
3	Lateral borders of pronotum flattened and stretched laterally (Fig. 1: 13–14; Fig. 2: 4–5)	4
–	Lateral borders of pronotum unlike above (Fig. 1: 4–5) (*Scleropatrum* Reitter, 1887)	*Scleropatrum horridum horridum* Reitter, 1898
4	Metathoracic wing sheath incompletely shrouded by elytral sheath (Fig. 1: 10–12) (*Gonocephalum* Solier, 1834)	*Gonocephalum reticulatum* Motschulsky, 1854
–	Metathoracic wing sheath completely shrouded by elytral sheath (Fig. 2: 1–3) (*Opatrum* Fabricius, 1775)	*Opatrum (Opatrum) subaratum* Faldermann, 1835
5	Anterior border of pronotum broadly emarginated and the emargination straight in middle, posterior angles prominent (Fig. 2: 13)	*Eumylada potanini* (Reitter, 1889)
–	Anterior border of pronotum broadly emarginated and the emargination protuberant in middle, posterior angles not prominent (Fig. 3: 4)	*Eumylada punctifera* (Reitter, 1889)
6	BUL longer than UL. (Fig. 3: 15–16; Fig. 4: 6–7) (*Penthicus* Faldermann, 1836)	7
–	BUL shorter than UL.(Fig. 4: 15–16; Fig. 5: 6–7, 15–16; Fig. 6: 6–7)	8
7	Anterior border of pronotum broadly emarginated and the emargination distinctly protuberant in middle (Fig. 3: 13)	*Penthicus (Myladion) alashanicus* (Reichardt, 1936)
–	Anterior border of pronotum broadly emarginated and the emargination straight in middle (Fig. 4: 4)	*Penthicus (Myladion) nojonicus* (Kaszab, 1968)
8	Pronotum nearly oval, with lateral borders broadly protuberant. (Fig. 5: 4, 13; Fig. 6: 4) (*Melanesthes* Lacordaire, 1859)	9
–	Pronotum subquadrate, with lateral borders nearly straight (Fig. 4: 13) (*Myladina* Reitter, 1889)	*Myladina unguiculina* Reitter, 1889
9	Urogomphi parallel to and slightly separated from each other (Fig. 5: 15–16; Fig. 6: 6–7)	10
–	Urogomphi diverging posteriorly and distinctly separated from each other (Fig. 5: 6–7)	*Melanesthes (Opatronesthes) rugipennis* Reitter, 1889
10	Urogomphi with apices leaning against each other (Fig. 5: 15–16)	*Melanesthes *(*Melanesthes*) *maxima maxima* Ménétriès, 1854
–	Urogomphi with apices slightly separated from each other (Fig. 6: 6–7)	*Melanesthes (Melanesthes) jintaiensis* Ren, 1992

### (1) Genus *Scleropatrum* Reitter, 1887

#### Scleropatrum horridum horridum

Reitter, 1898

http://species-id.net/wiki/Scleropatrum_horridum_horridum

Fig. 1: 1–9

##### Redescription.

Male. Body covered with short setae on surface; the total number of setose tubercles and setae on vertex, mandibles, the last segment of maxillary palpus, pronotal borders and hypomeron about 40–50 and 40–48, respectively.

Head bent at right angle to pronotum. Labrum covered with short setae along anterior and lateral borders, anterior border straight. Oculus reniform.

Pronotum: transverse, widest near middle, with the anterior part distinctly narrowed and the posterior part slightly narrowed, anterior border broadly emarginated and the emargination slightly protuberant in middle, lateral borders broadly protuberant; posterior border of pronotum bisinuate (Fig. 1: 4); anterior angles acute, posterior angles nearly right-angled; disc flat, sparsely covered with setae, with short and narrow furrows.

Mesonotum slightly convex in middle of posterior part, posterior border protuberant. Posterior border of metanotum straight.

Elytral sheath striped and sparsely setose. Metathoracic wing sheath completely shrouded by elytral sheath (Fig. 1: 1–3).

Abdominal segments I–IX curved ventrally. The width of abdominal segments I–VII gradually narrowed posteriorly. Posterior border of abdominal sternite VIII emarginated in middle, each side of the emargination with a long seta. Posterior border of abdominal tergite IX emarginated in middle; urogomphi diverging from each other, BUL subequal to UL (Fig. 1: 6–7). Abdominal tergites I–VII each with a concaveness between its lateral process and lateral border in middle, lateral processes each with 2 large and several minute setose tubercles along outer border, of which 1–2 minute setose tubercles present in front of large setose tubercle I, 2–4 minute setose tubercles between large setose tubercles I and II, 3–4 minute setose tubercles behind large setose tubercle II (Fig. 1: 8–9). Outer borders of lateral processes of abdominal tergites I–VI straight. Spiracles of abdominal tergites I–VI oval, slightly convex.

Gonotheca without apophysis.

Female. Similar to the male, but posterior border of abdominal sternite VIII broadly protuberant; gonotheca with an obtusely rounded apophysis on the anterior part and a conical apophysis on the posterior part.

##### Measurements.

BL: 10–13 mm; PL: 2.7–3.5 mm; PW: 3.8–4.4 mm; MSL: 0.8–0.9 mm; MTL: 0.6–0.8 mm; UL: 0.8–1.3 mm; BUL: 1.1–1.3 mm.

##### Material examined.

adults collected information: 1 May 2002, Shutai Town, Haiyuan County, Ningxia, China; pupation date: 17 May 2003; adults collected information: 10 July 2001, Suyukou, Helan Mountain, Ningxia, China; pupation date: 28 June 2002.

##### Remarks.

This species was included in the key of tenebrionid pupae by Yu et al. (1999), but only mentioned with some characters for identification. Here its morphological characters are described in detail and provided with the photos for the first time.

### (2) Genus *Gonocephalum* Solier, 1834

#### Gonocephalum reticulatum

Motschulsky

http://species-id.net/wiki/Gonocephalum_reticulatum

Fig. 1: 10–18

##### Redescription.

Male. Body sparsely covered with short setae; the total number of setose tubercles and setae on pronotal borders, hypomeron, vertex, mandibles and the last segment of maxillary palpus about 14–18 and 22–28, respectively.

Head bent at acute angle to pronotum, each side with a longitudinal, shallow groove between clypeus and gena. Labrum without setae, anterior border rounded. Oculus oval.

Pronotum: transverse, widest near middle, with the anterior part distinctly narrowed and the posterior part slightly narrowed; anterior border broadly emarginated and the emargination straight in middle; lateral borders broadly protuberant, flattened and stretched laterally; posterior border of pronotum bisinuate (Fig. 1: 13); anterior angles acute, posterior angles obtuse; disc flat, without setae, with short and narrow furrows.

Mesonotum slightly convex in middle of posterior part, posterior border protuberant. Posterior border of metanotum straight.

Elytral sheath striped and sparsely setose. Metathoracic wing sheath incompletely shrouded by elytral sheath (Fig. 1: 10–12).

Abdominal segments III–IX curved ventrally. The width of abdominal segments I–VII subequal to each other. Posterior border of abdominal sternite VIII slightly emarginated in middle, each side of the emargination with a long seta. Posterior border of abdominal tergite IX emarginated in middle; urogomphi diverging from each other, BUL subequal to UL (Fig. 1: 15–16). Abdominal tergites I–VII each with a concaveness between its lateral process and lateral border in middle, lateral processes each with 2 equally large and several minute setose tubercles along outer border, of which no minute setose tubercle presents in front of large setose tubercles I, 1 minute setose tubercle between large setose tubercles I and II and behind II, respectively (Fig. 1: 17–18). Outer borders of lateral processes of abdominal tergites I–VI straight. Spiracles of abdominal tergites I–VI nearly rounded, slightly convex.

Gonotheca without apophysis.

Female. Similar to the male, but posterior border of abdominal sternite VIII broadly protuberant; gonotheca without apophysis on the anterior part but with a conical apophysis on the posterior part.

##### Measurements.

BL: 5–5.5 mm; PL: 1.4–1.5 mm; PW: 2–2.1 mm; MSL: 0.9–1.0 mm; MTL: 1.1–1.2 mm; UL: 0.5 mm; BUL: 0.5 mm.

##### Material examined.

3, adults collected information: 6 April 1999, Ningxia Agriculture College, Yinchuan City, Ningxia, China; pupation date: 20 June 1999.

##### Remarks.

This species was included in the key of tenebrionid pupae by Yu et al. (1999), but only mentioned with some characters for identification. Here its morphological characters are described in detail and provided with the photos for the first time.

### (3) Genus *Opatrum* Fabricius, 1775

#### Opatrum (Opatrum) subaratum

Faldermann, 1835

http://species-id.net/wiki/Opatrum_subaratum

Fig. 2: 1–9

##### Redescription.

Male. Body nearly glabrous. Pronotal borders with 8–10 setose tubercles.

Head bent at acute angle to pronotum. Anterior border of labrum slightly emarginated in middle. Vertex with a central apophysis, in front of the apophysis with a pair of pits, behind the apophysis with a transverse, shallow groove. Oculus oval.

**Figure 2.**
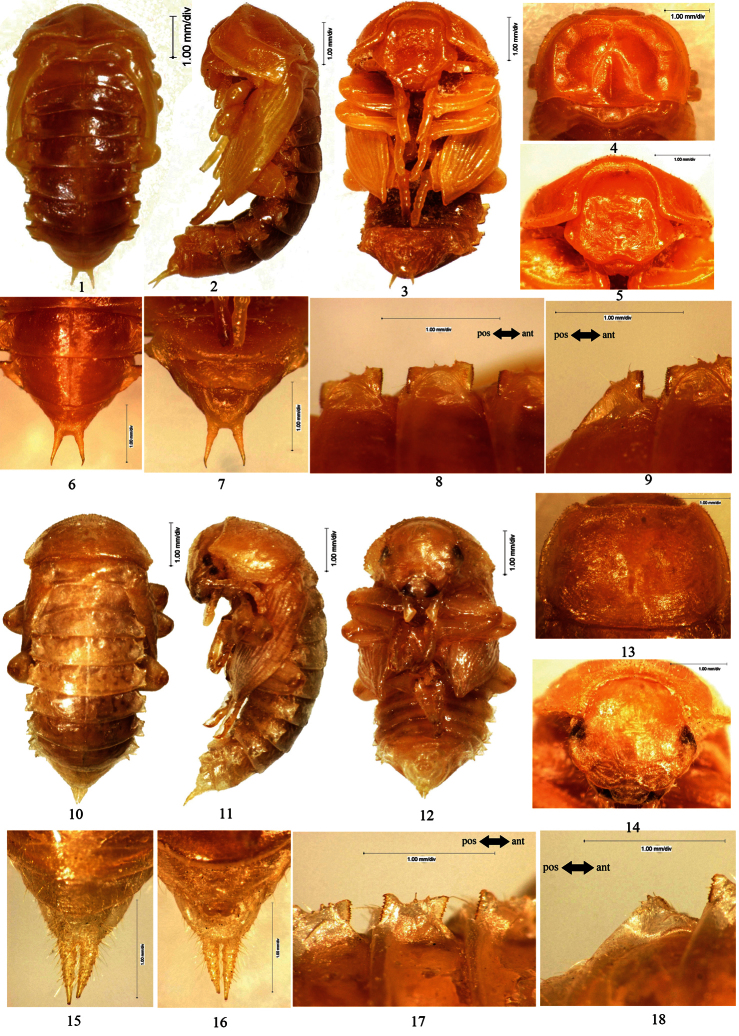
**1–9** *Opatrum (Opatrum) subaratum* Faldermann, 1835 **1** Pupal habitus in dorsal view **2** Pupal habitus in lateral view **3** Pupal habitus in ventral view **4** Pronotum **5** Head **6** Urogomphi in dorsal view **7 **Urogomphi in ventral view **8** Lateral process of abdominal tergite V **9** Lateral process of abdominal tergite VII **10–18** *Eumylada potanini* (Reitter, 1889) **10** Pupal habitus in dorsal view **11** Pupal habitus in lateral view **12** Pupal habitus in ventral view **13** Pronotum **14** Head **15** Urogomphi in dorsal view **16** Urogomphi in ventral view **17** Lateral process of abdominal tergite V **18** Lateral process of abdominal tergite VII.

Pronotum: transverse, widest near middle, with the anterior part distinctly narrowed and the posterior part slightly narrowed; anterior border broadly emarginated and the emargination straight in middle; lateral borders broadly protuberant, flattened and stretched laterally (Fig. 2: 4–5); posterior border bisinuate (Fig. 2: 4); a longitudinal groove extended along dorsomeson from middle to posterior border; anterior angles obtuse, posterior angles acute; disc convex, glabrous, with short and narrow furrows.

Mesonotum slightly convex in middle of posterior part, posterior border protuberant. Posterior border of metanotum straight.

Elytral sheath striped and sparsely setose. Metathoracic wing sheath completely shrouded by elytral sheath (Fig. 2: 1–3).

Abdominal segments III–IX curved ventrally. The width of abdominal segments I–VII gradually narrowed posteriorly. Posterior border of abdominal sternite VIII slightly emarginated in middle, each side of the emargination with a long seta. Posterior borders of abdominal tergite IX straight; urogomphi diverging from each other, BUL shorter than UL (Fig. 2: 6–7). Abdominal tergites I–VII each with a concaveness between its lateral process and lateral border in middle, lateral processes each with 2 equally large and several minute setose tubercles along outer border, of which no minute setose tubercle presents in front of large setose tubercles I, 1–2 minute setose tubercles between large setose tubercles I and II and behind II, respectively (Fig. 2: 8–9). Lateral processes with anterior and posterior borders slightly sclerotized, outer borders of lateral processes of abdominal tergites I–VI slightly protuberant. Spiracles of abdominal tergites I–VI oval.

Gonotheca without apophysis.

Female. Unknown.

##### Measurements.

BL: 6.7 mm; PL: 2.2 mm; PW: 3.5 mm; MSL: 0.5 mm; MTL: 0.6 mm; UL: 0.7 mm; BUL: 0.6 mm.

##### Material examined.

2, adults collected information: 30 June 1998, Xuanhua County, Hebei, China; pupation date: 22–26 Septemper 2000.

##### Remarks.

This species was included in the key of tenebrionid pupae by Yu et al. (1999), but only mentioned with some characters for identification. Here its morphological characters are described in detail and provided with the photos for the first time.

### (4) Genus *Eumylada* Reitter, 1889

#### Eumylada potanini

(Reitter, 1889)

http://species-id.net/wiki/Eumylada_potanini

Fig. 2: 10–18

##### Description.

Male. Body covered with setae on surface. Pronotal borders, hypomeron, vertex, labrum, mandibles, the last segment of maxillary palpus and abdominal sternite with dense, short setae.

Head bent at right angle to pronotum. Anterior border of labrum slightly emarginated in middle. Oculus reniform, densely covered with setae on posterior part.

Pronotum: transverse, widest at posterior one-fourth, with the anterior part distinctly narrowed and the posterior part slightly narrowed; anterior border broadly emarginated and the emargination straight in middle, lateral borders broadly protuberant, posterior border bisinuate (Fig. 2: 13); anterior angles acute, posterior angles slightly obtuse, protruding; disc slightly convex, sparsely covered with setae, with short and narrow furrows.

Mesonotum slightly convex in middle of posterior part, posterior border slightly protuberant. Posterior border of metanotum straight.

Elytral sheath striped, setose. Each elytral sheath with an apophysis near base. Metathoracic wing sheath completely shrouded by elytral sheath (Fig. 2: 10–12).

Abdominal segments I–IX curved ventrally. The width of abdominal segments I–VII gradually narrowed posteriorly. Abdominal tergites I–III each with 2 pits near anterior border and placed on both sides of dorsomeson, the pits of tergite I obscure, those of tergites II and III distinct. Posterior border of abdominal sternite VIII distinctly emarginated in middle, each side of the emargination with a long seta. Urogomphi parallel to and distinctly separated from each other, BUL shorter than UL (Fig. 2: 15–16). Abdominal segment IX and urogomphi densely covered with long setae. Abdominal tergites I–VII each with a concaveness between its lateral process and lateral border in middle, lateral processes thin, nearly transparent, each with 2 large setose tubercles and 0–2 setae along outer border, without minute setose tubercle (Fig. 2: 17–18). Outer borders of lateral processes of abdominal tergites I–VI slightly emarginated in middle. Spiracles of abdominal tergites I–VI oval.

Gonotheca without apophysis.

Female. Unknown.

##### Measurements.

BL: 6.9 mm, PL: 2.2 mm; PW: 3.4 mm; MSL: 0.7 mm; MTL: 0.7 mm; UL: 0.7 mm; BUL: 0.1 mm.

##### Material examined.

adults collected information: 19 April 2002, Baijitan, Lingwu County, Ningxia, China; pupation date: 22 May 2003.

#### Eumylada punctifera

(Reitter, 1889)

http://species-id.net/wiki/Eumylada_punctifera

Fig. 3: 1–9

##### Redescription.

Female. Body densely covered with fine setae on surface, except sprasely on vertex, pronotum, tibiae and tarsus; the total number of setose tubercles and setae on pronotal borders, hypomeron, vertex, mandibles and the last segment of maxillary palpus about 56 and 82, respectively.

Head bent at right angle to pronotum. Anterior border of labrum rounded. Oculus reniform.

**Figure 3.**
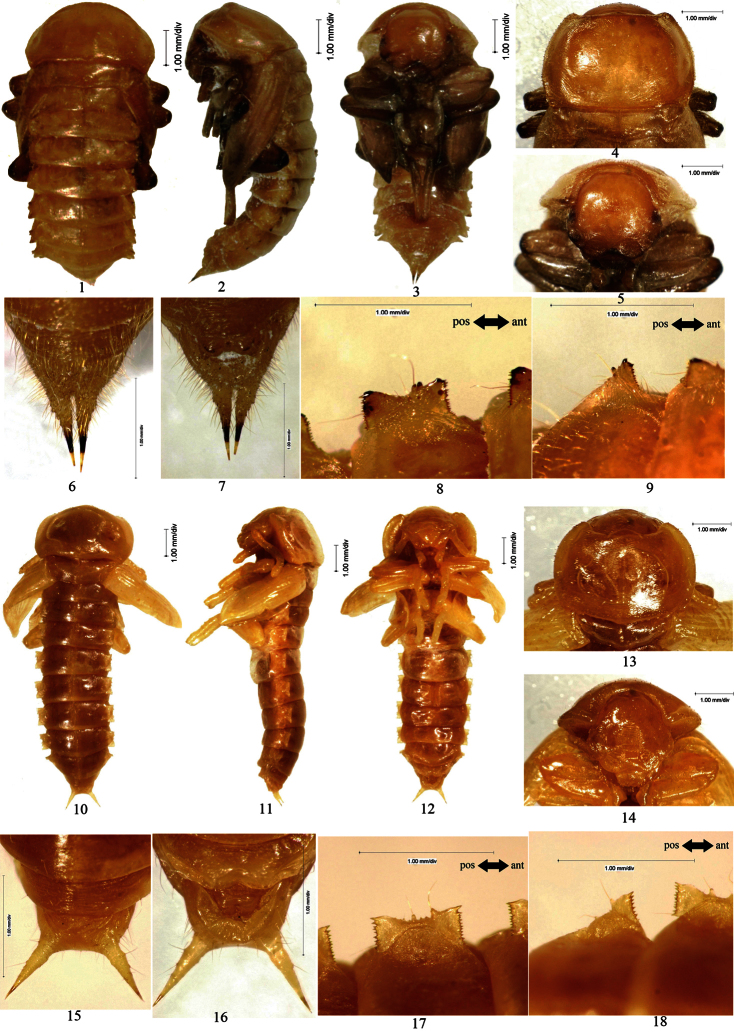
**1–9** *Eumylada punctifera* (Reitter, 1889) **1**﻿Pupal habitus in dorsal view **2** Pupal habitus in lateral view **3** Pupal habitus in ventral view **4** Pronotum **5** Head **6** Urogomphi in dorsal view **7** Urogomphi in ventral view **8** Lateral process of abdominal tergite V **9** Lateral process of abdominal tergite VII **10–18** *Penthicus (Myladion) alashanicus* (Reichardt, 1936) **10** Pupal habitus in dorsal view **11** Pupal habitus in lateral view **12** Pupal habitus in ventral view **13** Pronotum **14** Head **15** Urogomphi in dorsal view **16** Urogomphi in ventral view **17** Lateral process of abdominal tergite V **18** Lateral process of abdominal tergite VII.

Pronotum: transverse, widest near middle, with the anterior part distinctly narrowed and the posterior part slightly narrowed; anterior border broadly emarginated and the emargination straight in middle, lateral borders broadly protuberant, posterior border bisinuate (Fig. 3: 4); both anterior and posterior angles acute; disc slightly convex, with short and narrow furrows.

Mesonotum slightly convex in middle of posterior part, posterior border protuberant. Posterior border of metanotum slightly protuberant.

Elytral sheath striped and sparsely setose. Metathoracic wing sheath completely shrouded by elytral sheath (Fig. 3: 1–3).

Abdominal segments III–IX curved ventrally. The width of abdominal segments I–VII gradually narrowed posteriorly. Posterior border of abdominal sternite VIII broadly protuberant; abdominal segment IX and urogomphi densely covered with long setae. Urogomphi parallel to and distinctly separated from each other, BUL shorter than UL (Fig. 3: 6–7). Abdominal tergites I–VII each with a concaveness between its lateral process and lateral border in middle, lateral processes each with 3 large setose tubercles and 6–10 setae along outer border, without minute setose tubercle (Fig. 3: 8–9). Outer borders of lateral processes of abdominal tergites I–VI slightly emarginated in middle. Spiracles of abdominal tergites I–VI oval.

Gonotheca with an obtusely rounded apophysis on the anterior part and a conical apophysis on the posterior part.

Male. Unknown.

##### Measurements.

BL: 7.9 mm; PL: 2.4 mm; PW: 3.8 mm; MSL: 0.7 mm; MTL: 0.7 mm; UL: 0.5 mm; BUL shorter than 0.1mm.

##### Material examined.

adults collected information: 2 May 2000, Shijiazi Village, Gulang County, Gansu, China; pupation date: 14 June 2001.

##### Remarks.

This species was included in the key of tenebrionid pupae by Yu, Ren and Dai (1999), but only mentioned with some characters for identification. Here its morphological characters are described in detail and provided with the photos for the first time.

### (5) Genus *Penthicus* Faldermann, 1836

#### Penthicus (Myladion) alashanicus

(Reichardt, 1936)

http://species-id.net/wiki/Penthicus_alashanicus

Fig. 3: 10–18

##### Redescription.

Male. Body sparsely covered with short setae on surface of pronotum, abdominal segments and elytral sheath. The total number of setose tubercles and setae on pronotal borders, hypomeron, vertex, mandibles and the last segment of maxillary palpus about 26–30 and 24–32, respectively.

Head bent at acute angle to pronotum, each side with an obscure groove between clypeus and gena. Anterior border of labrum distinctly emarginated in middle. Oculus oval.

Pronotum: transverse, widest near middle, with the anterior part distinctly narrowed and the posterior part slightly narrowed; anterior border broadly emarginated and the emargination distinctly protuberant in middle; lateral borders broadly protuberant, posterior border broadly protuberant (Fig. 3: 13); anterior angles acutely rounded, posterior angles obtusely sharp; disc flat, with short and narrow furrows.

Mesonotum slightly convex in middle of posterior part, posterior border protuberant. Posterior border of metanotum straight.

Elytral sheath striped and sparsely setose. Metathoracic wing sheath incompletely shrouded by elytral sheath (Fig. 3: 10–12).

Abdominal segments VI–IX curved ventrally. The width of abdominal segments I–VI subequal to each other. Posterior border of abdominal sternite VIII emarginated in middle, each side of the emargination with a long seta. Posterior border of abdominal tergite IX broadly emarginated in middle; urogomphi diverging from each other, BUL longer than UL (Fig. 3: 15–16). Abdominal tergites I–VII each with a concaveness between its lateral process and lateral border in middle, lateral processes each with 2 equally large and several minute setose tubercles along outer border, of which 0–1 minute setose tubercle presents in front of large setose tubercle I, 2 minute setose tubercles between large setose tubercles I and II, 3 minute setose tubercles behind large setose tubercle II (Fig. 3: 17–18). Outer borders of lateral processes of abdominal tergites I–VI slightly emarginated in middle. Spiracles of abdominal tergites I–VI nearly rounded, slightly convex.

Gonotheca without apophysis.

Female. Unknown.

##### Measurements.

BL: 11.0 mm; PL: 2.7 mm; PW: 3.7 mm; MSL: 0.9 mm; MTL: 0.7 mm; UL: 0.7 mm; BUL: 1.4 mm.

##### Material examined.

adults collected information: 7 June 1999, Longshou Moutain, Shandan County, Gansu, China; pupation date: 31 Auguest 1999.

##### Remarks.

This species was included in the key of tenebrionid pupae by Yu et al. (1999) , but only mentioned with some characters for identification. Here its morphological characters are described in detail and provided with the photos for the first time.

#### Penthicus (Myladion) nojonicus

(Kaszab, 1968)

http://species-id.net/wiki/Penthicus_ nojonicus

Fig. 4: 1–9

##### Redescription.

Female. Body covered with short setae on surface, of which slightly densely on head, pronotum and abdominal sternite; the total number of setose tubercles and setae on pronotal borders, hypomeron, vertex, mandibles and the last segment of maxillary palpus about 30–34 and 40–50, respectively.

Head bent at acute angle to pronotum. Labrum sparsely covered with short setae along anterior and lateral borders, anterior border slightly emarginated in middle. Oculus reniform.

**Figure 4.**
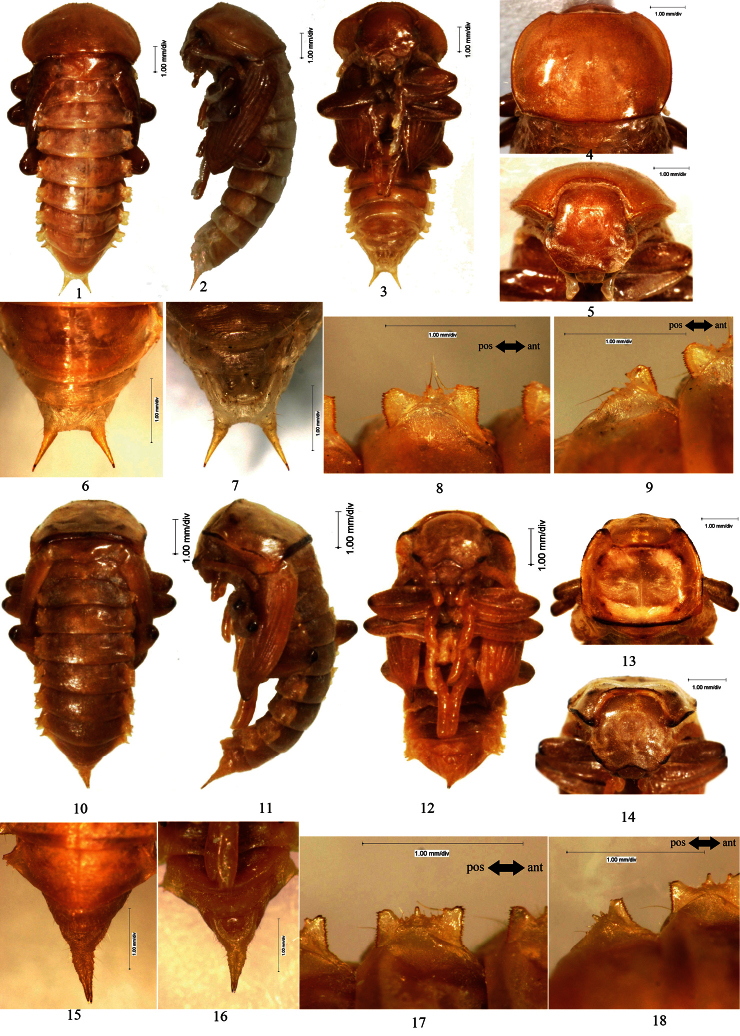
**1–9** *Penthicus (Myladion) nojonicus* (Kaszab, 1968) **1** Pupal habitus in dorsal view **2** Pupal habitus in lateral view **3** Pupal habitus in ventral view **4** Pronotum **5** Head **6** Urogomphi in dorsal view **7 **Urogomphi in ventral view **8** Lateral process of abdominal tergite V **9** Lateral process of abdominal tergite VII **10–18** *Myladina unguiculina* Reitter, 1889 **10** Pupal habitus in dorsal view **11** Pupal habitus in lateral view **12** Pupal habitus in ventral view **13** Pronotum **14** Head **15** Urogomphi in dorsal view **16** Urogomphi in ventral view **17** Lateral process of abdominal tergite V **18** Lateral process of abdominal tergite VII.

Pronotum: transverse, widest near middle, the anterior part narrowed almost the same as the posterior part; anterior border broadly emarginated and the emargination straight in middle, lateral and posterior borders broadly protuberant, posterior border slightly emarginated in middle (Fig. 4: 4); anterior angles acute, posterior angles obtusely acute; disc flat, glabrous, with short and narrow furrows.

Mesonotum slightly convex in middle of posterior part, posterior border slightly protuberant. Posterior border of metanotum straight.

Elytral sheath striped and sparsely setose. Metathoracic wing sheath completely shrouded by elytral sheath (Fig. 4: 1–3).

Abdominal segments I–IX curved ventrally. The width of abdominal segments I–VII gradually narrowed posteriorly. Posterior border of abdominal sternite VIII broadly protuberant. Posterior border of abdominal tergite IX straight; urogomphi diverging from each other, BUL longer than UL (Fig. 4: 6–7). Abdominal tergites I–VII each with a concaveness between its lateral process and lateral border in middle, lateral processes each with 2 large setose tubercles and several minute setose tubercles along outer border, of which no minute setose tubercle presents in front of large setose tubercles I, 1–2 minute setose tubercles between large setose tubercles I and II, 2–3 minute setose tubercles behind large setose tubercle II (Fig. 4: 8–9). Outer borders of lateral processes of abdominal tergites I–VI emarginated in middle. Spiracles of abdominal tergites I–VI oval, slightly convex.

Gonotheca with an obtusely rounded apophysis on the anterior part and a conical apophysis on the posterior part.

Male. Unknown.

##### Measurements.

BL: 11.0 mm; PL: 3.4 mm; PW: 4.6 mm; MSL: 0.9 mm; MTL: 0.9 mm; UL: 0.8 mm; BUL: 1.3 mm.

##### Material examined.

adults collected information: 14 July 2000, Fanjiayao Village, Baiyin City, Gansu, China; pupation date: 14 June 2001.

##### Remarks.

This species was included in the key of tenebrionid pupae by Yu et al. (1999), but only mentioned with some characters for identification. Here its morphological characters are described in detail and provided with the photos for the first time.

### (6) Genus *Myladina* Reitter, 1889

#### Myladina unguiculina

Reitter, 1889

http://species-id.net/wiki/Myladina_unguiculina

Fig. 4: 10–18

##### Redescription.

Male. Body covered with setae on surface, of which slightly densely on elytral sheath and abdominal segments; the total number of setose tubercles and setae on pronotal borders, hypomeron, vertex, mandibles and the last segment of maxillary palpus about 14–20 and 40–60, respectively.

Head bent at right angle to pronotum. Labrum sparsely covered with setae along anterior and lateral borders, anterior border distinctly emarginated in middle. Oculus reniform.

Pronotum: subquadrate, widest near middle, with the anterior part distinctly narrowed and the posterior part slightly narrowed; anterior border broadly emarginated and the emargination slightly protuberant in middle, lateral borders nearly straight, posterior border straight (Fig. 4: 13); anterior angles acutely rounded, posterior angles acute; disc flat, glabrous, with short and narrow furrows.

Mesonotum slightly convex in middle of posterior part, posterior border protuberant. Posterior border of metanotum straight.

Elytral sheath striped and densely setose. Metathoracic wing sheath completely shrouded by elytral sheath (Fig. 4: 10–12).

Abdominal segments I–IX curved ventrally. The width of abdominal segments I–VII gradually narrowed posteriorly. Posterior border of abdominal sternite VIII emarginated in middle, each side of the emargination with a long seta. Abdominal segment IX, urogomphi and gonotheca densely covered with setae. Urogomphi broad at base, parallel to each other, with apices slightly separated, BUL much shorter than UL (Fig. 4: 15–16). Abdominal tergites I–VII each with a concaveness between its lateral process and lateral border in middle, lateral processes each with 2 large and several minute setose tubercles along outer border, large setose tubercles II distinctly larger than I, no minute setose tubercle presents in front of large setose tubercles I, 2–3 minute setose tubercles between large setose tubercles I and II and behind II, respectively (Fig. 4: 17–18). Outer borders of lateral processes of abdominal tergites I–VI slightly protuberant in middle. Spiracles of abdominal tergites I–VI oval.

Gonotheca without apophysis.

Female. Similar to the male, but posterior border of abdominal sternite VIII broadly protuberant; gonotheca with an obtusely rounded apophysis on the anterior part and a conical apophysis on the posterior part.

**Measurements.** BL: 7.5–7.7 mm; PL: 2.6–2.7 mm; PW: 3.3–3.5 mm; MSL: 0.8 mm; MTL: 0.5 mm; UL: 0.8–0.9 mm; BUL: 0.1 mm.

**Material examined.** 10, Adults collected information: 24 June 2001, Gaoshawo Town, Yanchi County, Ningxia, China; pupation date: 2–10 September 2001.

**Remarks.** This species was included in the key of tenebrionid pupae by Yu et al. (1999), but only mentioned with some characters for identification. Here its morphological characters are described in detail and provided with the photos for the first time.

### (7) Genus *Melanesthes* Lacordaire, 1859

#### Melanesthes (Opatronesthes) rugipennis

Reitter, 1889

http://species-id.net/wiki/Melanesthes_rugipennis

Fig. 5: 1–9

##### Description.

Male. Body covered with setae on surface, of which slightly dense on abdominal sternite, head and pronotum; the total number of setose tubercles and setae on pronotal borders, hypomeron, vertex, mandibles and the last segment of maxillary palpus about 22–26 and 10–16, respectively.

Head bent at acute angle to pronotum, densely covered with setae between oculus and pronotum. Labrum covered with short setae along anterior border, anterior border distinctly emarginated in middle. Oculus reniform.

**Figure 5.**
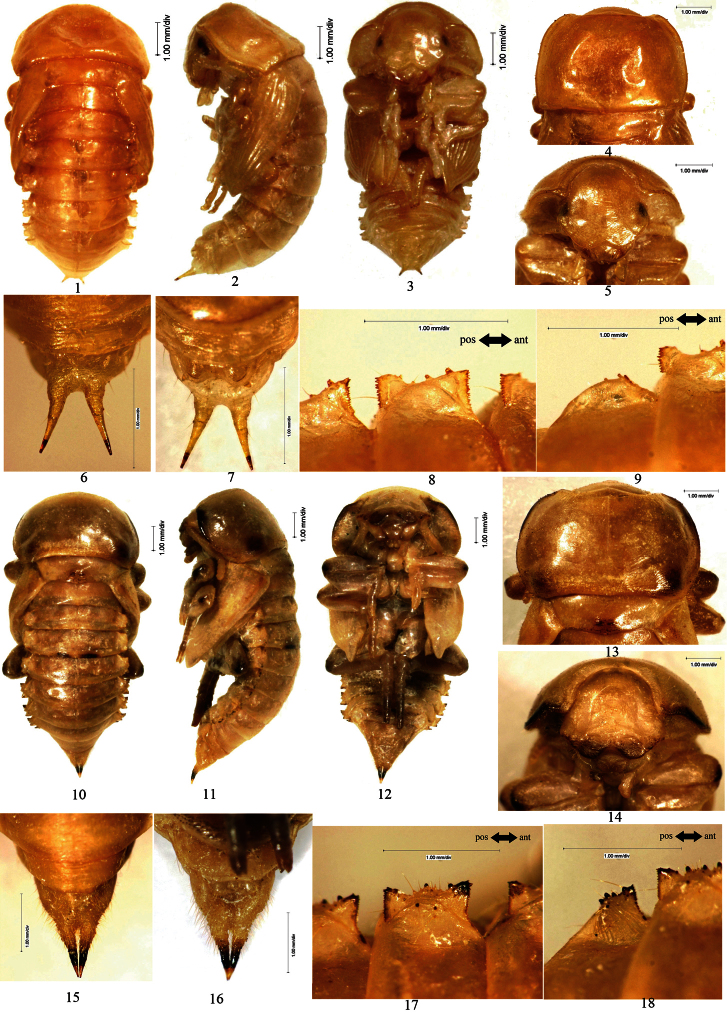
**1–9** *Melanesthes (Opatronesthes) rugipennis* Reitter, 1889 **1** Pupal habitus in dorsal view **2 **Pupal habitus in lateral view **3** Pupal habitus in ventral view **4** Pronotum **5** Head **6** Urogomphi in dorsal view **7** Urogomphi in ventral view **8** Lateral process of abdominal tergite V **9** Lateral process of abdominal tergite VII **10–18** *Melanesthes (Melanesthes) maxima maxima* Ménétriès, 1854 **10** Pupal habitus in dorsal view **11** Pupal habitus in lateral view **12** Pupal habitus in ventral view **13** Pronotum **14** Head **15 **Urogomphi in dorsal view **16** Urogomphi in ventral view **17** Lateral process of abdominal tergite V **18** Lateral process of abdominal tergite VII.

Pronotum: transverse, widest at posterior one-fourth, with the anterior part distinctly narrowed and the posterior part slightly narrowed; anterior border broadly emarginated and the emargination protuberant in middle, lateral borders broadly protuberant, posterior border straight (Fig. 5: 4); anterior angles acute, posterior angles nearly right-angled, acute; disc flat, sparsely covered with setae, with short and narrow furrows.

Mesonotum slightly convex in middle of posterior part, posterior border straight. Posterior border of metanotum slightly protuberant.

Elytral sheath striped and glabrous. Metathoracic wing sheath completely shrouded by elytral sheath (Fig. 5: 1–3).

Abdominal segments I–IX curved ventrally. The width of abdominal segments I–VII gradually narrowed posteriorly. Posterior border of abdominal sternite VIII emarginated in middle, each side of the emargination with a long seta. Abdominal sternite IX with 8–10 setae. Posterior border of abdominal tergite IX emarginated in middle. Urogomphi diverging from each other, each with a long seta on outer border , BUL shorter than UL (Fig. 5: 6–7). Abdominal tergites I–VII each with a concaveness between its lateral process and lateral border in middle, lateral processes each with 2 large and several minute setose tubercles along outer border, of which no minute setose tubercle presents in front of large setose tubercles I, 1 minute setose tubercle between large setose tubercles I and II, 1–2 minute setose tubercles behind large setose tubercle II (Fig. 5: 8–9). Outer borders of lateral processes of abdominal tergites I–VI slightly emarginated in middle. Spiracles of abdominal tergites I–VI oval, slightly convex.

Gonotheca without apophysis.

Female. Similar to the male, but posterior border of abdominal sternite VIII broadly protuberant; gonotheca with an obtusely rounded apophysis on the anterior part and a conical apophysis on the posterior part.

##### Measurements.

BL: 8.5–8.6 mm; PL: 3.0–3.1 mm; PW: 4.3–4.4 mm; MSL: 0.9 mm; MTL: 0.8 mm; UL: 0.7–0.8 mm; BUL: 0.6–0.7 mm.

##### Material examined.

3, adults collected information: 1 May 2002, Shutai Town, Haiyuan County, Ningxia, China; pupation date: 25–27 May 2003.

#### Melanesthes (Melanesthes) maxima maxima

Ménétriès, 1854

http://species-id.net/wiki/Melanesthes_maxima_maxima

Fig. 5: 10–18

##### Redescription.

Female. Body covered with setae on surface; setae on abdominal tergites and urogomphi more; the total number of setose tubercles and setae on pronotal borders, hypomeron, vertex, mandibles and the last segment of maxillary palpus about 16–20 and 50–58, respectively.

Head bent at acute angle to pronotum. Labrum covered with sparsely short setae along anterior and lateral borders, anterior border distinctly emarginated in middle. Oculus oval.

Pronotum: transverse, widest near middle, with the anterior part distinctly narrowed and the posterior part slightly narrowed; anterior border broadly emarginated and the emargination protuberant in middle, lateral borders broadly protuberant, posterior border straight (Fig. 5: 13); anterior angles rounded, posterior angles obtusely rounded; disc flat, glabrous, with short and narrow furrows, each side with a apophysis before the posterior border.

Mesonotum slightly convex in middle of posterior part, posterior border protuberant. Metanotum slightly convex in middle of anterior part, posterior border straight.

Elytral sheath obscurely striped and finely setose; with a distinct groove between elytral sheath and mesonotum. Metathoracic wing sheath completely shrouded by elytral sheath (Fig. 5: 10–12).

Abdominal segments III–IX curved ventrally. The width of abdominal segments I–VII gradually narrowed posteriorly. Posterior border of abdominal sternite VIII broadly protuberant. Abdominal segment IX and urogomphi densely covered with, brown, long setae. Urogomphi parallel to each other, with apices of urogomphi leaning against each other, BUL much shorter than UL (Fig. 5: 15–16). Abdominal tergites I–VII each with a concaveness between its lateral process and lateral border in middle, lateral processes each with 2–3 large setose tubercles and about 5–7 minute setose tubercles along outer border (Fig. 5: 17–18). Outer borders of lateral processes of abdominal tergites I–VI protuberant in middle. Spiracles of abdominal tergites I–VI oval.

Gonotheca with an obtusely rounded apophysis on the anterior part and a conical apophysis on the posterior part, a brown apophysis between gonothecas.

Male. Unkown.

##### Measurements.

BL: 11.8 mm; PL: 3.2 mm; PW: 5.4 mm; MSL: 0.3 mm; MTL: 0.2 mm; UL: 1.0 mm; BUL shorter than 0.1 mm.

##### Material examined.

adults collected information: 22 April 2001, Yaoba Town, Alxa Left Banner, Neimenggu, China; pupation date: 22 June 2001.

##### Remarks.

This species was included in the key of tenebrionid pupae by Yu et al. (1999), but only mentioned with some characters for identification. Here its morphological characters are described in detail and provided with the photos for the first time.

#### Melanesthes (Melanesthes) jintaiensis

Ren, 1992

http://species-id.net/wiki/Melanesthes_jintaiensis

Fig. 6: 1–9

##### Description.

Female. Body densely covered with setae on surface, of which distinctly long on mandibles and oculus; the total number of setose tubercles and setae on pronotal borders, hypomeron, vertex, mandibles and the last segment of maxillary palpus about 40–42 and 300–350, respectively.

Head bent at acute angle to pronotum, densely covered with setae between oculus and pronotum. Labrum densely covered with short setae along anterior and lateral borders, anterior border rounded. The suture between labrum and clypeus indistinct. Oculus reniform.

**Figure 6.**
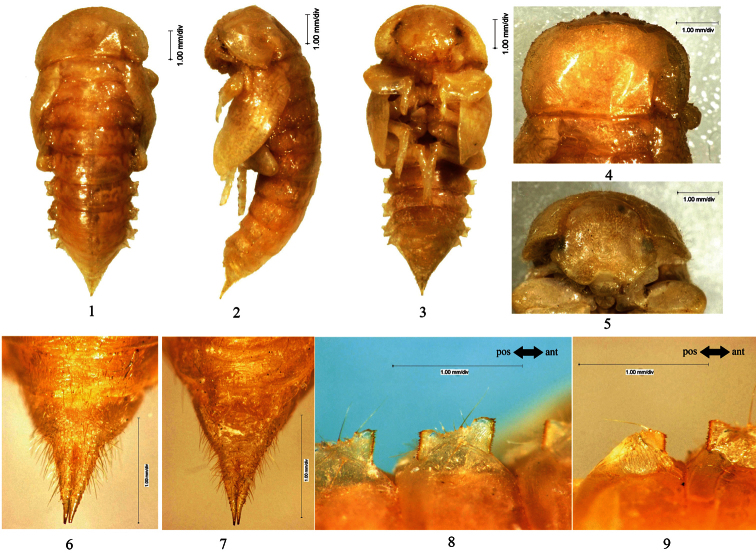
**1–9** *Melanesthes (Melanesthes) jintaiensis* Ren, 1992 **1** Pupal habitus in dorsal view **2** Pupal habitus in lateral view **3** Pupal habitus in ventral view **4** Pronotum **5** Head **6** Urogomphi in dorsal view **7** Urogomphi in ventral view **8** Lateral process of abdominal tergite V **9** Lateral process of abdominal tergite VII.

Pronotum: transverse, widest near middle, with the anterior part distinctly narrowed and the posterior part slightly narrowed; anterior border broadly emarginated and the emargination protuberant in middle, lateral borders broadly protuberant, posterior border straight (Fig. 6: 4); anterior angles acute, posterior angles rounded; disc flat, densely covered with setae, with short and narrow furrows.

Mesonotum slightly convex in middle of posterior part, posterior border slightly protuberant. Metanotum slightly convex in middle of anterior part, posterior border straight.

Elytral sheath striped and densely setose. Metathoracic wing sheath completely shrouded by elytral sheath (Fig. 6: 1–3).

Abdominal segments I–IX curved ventrally. The width of abdominal segments I–VII gradually narrowed posteriorly. Posterior border of abdominal sternite VIII broadly protuberant. Abdominal segment IX and urogomphi densely covered with brown, long setae. Urogomphi parallel to each other, with apices slightly separated from each other, BUL shorter than UL (Fig. 6: 6–7). Abdominal tergites I–VII each with a concaveness between its lateral process and lateral border in middle, lateral processes each with 2 large and several minute setose tubercles along outer border, of which no minute setose tubercle presents in front of large setose tubercles I, 1 minute setose tubercle between large setose tubercles I and II, 3–4 minute setose tubercles behind large setose tubercle II (Fig. 6: 8–9). Outer borders of lateral processes of abdominal tergites I–VI emarginated in middle. Spiracles of abdominal tergites I–VI oval, slightly convex.

Gonotheca with 6–8 setae, an obtusely rounded apophysis on the anterior part and a conical apophysis on the posterior part.

Male. Unknown.

##### Measurements.

BL: 9.5 mm; PL: 2.4 mm; PW: 4.1 mm; MSL: 0.9 mm; MTL: 0.8 mm; UL: 0.9 mm; BUL shorter than 0.1 mm.

##### Material examined.

adults collected information: 31 May 2002, Zhengshaqu Village, Yongning County, Ningxia, China; pupation date: 29 August 2002.

## Results and discussion

After describing in detail the eleven pupae from China, we studied and characterized the morphology of Opartini pupae. This was carried out by examining all available material as well as drawings and descriptions provided in the literature. The results indicate that all known Opartini pupae have a pair of urogomphi on abdominal tergite IX, the abdominal lateral processes are dorsoventrally flattened and bear strongly sclerotized, densely dentated anterior and posterior borders each.

Two main types of urogomphi can be identified in the pupae described in this paper, which may be a useful taxonomic character for distinguishing the genera. The urogomphi in *Scleropatrum horridum horridum*, *Gonocephalum reticulatum*, *Opatrum (Opatrum) subaratum*, *Penthicus (Myladion) alashanicus*, *Penthicus (Myladion) nojonicus*, *Melanesthes (Opatronesthes) rugipennis* are identical. In these species, the urogomphi are diverging from each other, but can be distinguished by the the relative value of length and distance between urogomphi. Comparatively, the urogomphi in *Eumylada potanini*, *Eumylada punctifera*, *Melanesthes (Melanesthes) maxima maxima*, *Melanesthes (Melanesthes) jintaiensis*, *Myladina unguiculina* are parallel to each other, but the species are different in if there is a space between them or not. Also, the pronotum is a good character to differentiate the species.

More taxonomic studies are needed in order to assess the value of different morphological characters, but the data presented here demonstrate that abdominal lateral processes, urogomphi and pronotum can be useful for the taxonomy of the pupal stages at geneic and specific levels within Opartini.

## Supplementary Material

XML Treatment for Scleropatrum horridum horridum

XML Treatment for Gonocephalum reticulatum

XML Treatment for Opatrum (Opatrum) subaratum

XML Treatment for Eumylada potanini

XML Treatment for Eumylada punctifera

XML Treatment for Penthicus (Myladion) alashanicus

XML Treatment for Penthicus (Myladion) nojonicus

XML Treatment for Myladina unguiculina

XML Treatment for Melanesthes (Opatronesthes) rugipennis

XML Treatment for Melanesthes (Melanesthes) maxima maxima

XML Treatment for Melanesthes (Melanesthes) jintaiensis
